# A Higher Neutrophil Count Is Associated with Favorable Achievement of Treatment-Free Remission in Patients with Chronic Myeloid Leukemia Who Received Second Generation Tyrosine Kinase Inhibitor as Frontline Treatment

**DOI:** 10.3390/clinpract14040097

**Published:** 2024-06-21

**Authors:** Hiroshi Ureshino, Yusuke Takeda, Kazuharu Kamachi, Takaaki Ono, Noriyoshi Iriyama, Eiichi Ohtsuka, Emiko Sakaida, Shinya Kimura

**Affiliations:** 1 Department of Hematology and Oncology, Research Institute for Radiation Biology and Medicine, Hiroshima University, 1-2-3, Kasumi, Minami-ku, Hiroshima 734-8553, Japan; 2 Next Generation Development of Genome and Cellular Therapy Program, Research Institute for Radiation Biology and Medicine (RIRBM), Hiroshima University, Hiroshima 734-8553, Japan; 3 Division of Hematology, Respiratory Medicine and Oncology, Department of Internal Medicine, Faculty of Medicine, Saga University, Saga 849-8501, Japan; sm2415@cc.saga-u.ac.jp (K.K.); shkimu@cc.saga-u.ac.jp (S.K.); 4 Department of Hematology, Chiba University Hospital, Chiba 260-8677, Japan; take-you@hospital.chiba-u.jp (Y.T.); esakaida@faculty.chiba-u.jp (E.S.); 5 Division of Hematology, Hamamatsu University School of Medicine, Shizuoka 431-3192, Japan; takaono@hama-med.ac.jp; 6 Division of Hematology and Rheumatology, Department of Medicine, Nihon University School of Medicine, Tokyo 173-8610, Japan; iriyama.noriyoshi@nihon-u.ac.jp; 7 Department of Hematology, Oita Prefectural Hospital, Oita 870-8511, Japan; e-ohtsuka@oitapref-hosp.jp

**Keywords:** chronic myeloid leukemia, treatment-free remission, tyrosine kinase inhibitors, neutrophil, cancer immunology

## Abstract

Background: ABL1 tyrosine kinase inhibitor discontinuation securely became among the therapeutic goal for chronic myeloid leukemia chronic phase patients (CML-CP). To establish successful prognostic factors for treatment-free remission (TFR), it is necessary to diagnose the patients with high-risk molecular relapse, however, a biomarker for the achievement of TFR has not been completely elucidated. Recent investigations have determined that neutrophils function crucially in cancer immunology. Patients and Methods: The research was a multicenter retrospective observational study to examine the correlation between TFR and neutrophil counts before TKI discontinuation. The investigation included patients having Philadelphia chromosome-positive CML-CP who attempted the discontinuation of TKIs after a durable deep molecular response between January 2012 and July 2021 at four institutions in Japan. Results: 118 CML-CP patients in total discontinued TKIs and an estimated 36-month TFR rate was 65.1%. 52 patients received second-generation TKIs as frontline. Higher neutrophil count (>3210/μL) at TKIs discontinuation was determined as an independent prognostic variable for TFR in patients who received second-generation TKIs as frontline [(HR, 0.235 (95%, confidence interval (CI) 0.078–0.711); *p* = 0.010]. Conclusions: The neutrophil-mediated immunomodulation can be a significant component for the effective achievement of TFR in CML supported by our clinical observation.

## 1. Introduction

The introduction of ABL1 tyrosine kinase inhibitors (TKIs) has provided a favorable life expectancy for patients with chronic myeloid leukemia (CML-CP) similar to that of the general populations [1]. However, late adverse events (e.g., cardiovascular events and renal dysfunction) and high-medical costs have become a new medical issue due to long-term TKIs treatment. Stop Imatinib (STIM) study which was a pioneering TKI discontinuation study revealed 41% of the patients who achieved a durable deep molecular response (DMR; complete molecular response at that time) for at least 2 years maintained treatment free remission (keeping DMR) [2]. The A-STIM study revealed a trigger for resuming imatinib after the loss of major molecular response (MMR) was sufficient, then the loss of MMR was defined as a molecular relapse after imatinib discontinuation [3]. Next, first-line or second-line second-generation TKI discontinuation studies were reported. The results were similar to the TKI discontinuation study with imatinib [4,5,6,7]. These TKI discontinuation studies indicated that approximately half of the patients who attained a durable deep molecular response (DMR) could halt TKIs without molecular relapse [2,3,4,5,6,7,8], hence TKI discontinuation became among the therapeutic goals for CML-CP patients [9]. To establish successful prognostic factors for TFR, it is necessary to determine the patients who are high-risk molecular relapses, still, biomarkers for the achievement of TFR have been incompletely elucidated due to a lack of comprehensive assessment to diagnose the prognostic factors for TFR in CML.

Longer TKI treatment duration [2,8,10], longer DMR duration [8,11], lower *BCR::ABL1* transcript levels [12,13], a lower Sokal score [2,4], higher natural killer (NK) cell counts at TKIs discontinuation [14,15], presence of withdrawal syndrome [10] may be a favorable prognosis factor for successful achievement of TFR. On the other hand, resistance to prior TKIs [4,5], e13a2 *BCR::ABL* transcript type [16] and higher regulatory T cell counts [17] may be associated with unfavorable prognostic factor.

Various shreds of evidence indicate that CML is sensitive to immunotherapy, including interferon α, allogeneic hematopoietic stem cell transplantation, and donor lymphocyte infusion, hence cancer immunosurveillance against CML is necessary to avert the relapse of patients with CML-CP. NK cells and T lymphocytes (T cells) are key components of the human immune system against viruses or cancers [18].

Recent studies have identified neutrophils also function critically in cancer immunology, although neutrophils are commonly known to function as antibacterial [19]. We earlier reported that higher neutrophil counts were a favorable prognostic factor for TFR in CML-CP patients [20]. The investigation was developed as a single center, retrospective analysis. We analyzed the association between neutrophil count and TFR outcome in patients with CML-CP.

## 2. Materials and Methods

### 2.1. Study Design and Patients

The research was a multicenter retrospective observational study to examine the correlation between TFR and neutrophil counts before TKI discontinuation. The investigation included patients having Philadelphia chromosome-positive CML-CP who attempted the discontinuation of TKIs (imatinib, dasatinib, nilotinib, or bosutinib) after a durable DMR between January 2012 and July 2021 at four institutions in Japan (Chiba University, Chiba, Japan; Hamamatsu University School of Medicine, Shizuoka, Japan; Nihon University School of Medicine, Tokyo, Japan; Oita Prefectural Hospital, Oita, Japan). CML was diagnosed following the World Health Organization classification of myeloid neoplasms and acute leukemia [21].

Baseline patient characteristics were obtained from hospital records such as general characteristics (age and sex), type of TKIs, laboratory data (complete white cell counts, neutrophil counts, and lymphocyte counts, molecular diagnosis (*BCR::ABL1* mRNA transcript level), and Sokal risk score. The final follow-up date was 31 July 2021.

The clinical research was approved by the Institutional Review Board of each participating hospital (2021-12-R-04)**.** Earlier published data was employed as control [20]. Two expert hematologists (HU and KK) reviewed all clinical data. All techniques involving human participants were undertaken following the principles of the Declaration of Helsinki. Informed consent was acquired by the opt-out method; information on the study, including the use of specimens objectives and the opportunity to opt-out, was made public, and no patients made objections.

### 2.2. Definition of Molecular Responses and Molecular Relapse

Molecular responses were defined based on *BCR::ABL1* mRNA transcript levels by real-time quantitative-PCR (RQ-PCR) applying the international scale. A major molecular response (MMR) was defined as a *BCR::ABL1* mRNA transcript level of ≤0.1%, MR^4.0^ was defined as ≤0.01%, MR^4.5^ was defined as ≤0.0032%, and undetectable minimal residual disease (UMRD) was defined as undetectable *BCR::ABL1* mRNA transcript level. DMR was defined as MR^4.0^ or a deeper response, and molecular relapse was determined as loss of the MMR.

### 2.3. Statistical Analysis

The cumulative incidence of TFR was calculated by the Kaplan-Meier method and differences were assessed using the log-rank test. Cox’s proportional hazard model was employed to examine the association between TFR and each of the variables (All variables (continuous variables were dichotomised at median values), and two-sided *p* values < 0.05 were considered statistically significant. Statistically significant differences between three or more groups or variables were determined using the one-way ANOVA and the Bonferroni was used as multiple pairwise tests. The Mann–Whitney U test was utilized to determine statistically significant differences between the two groups. A comparison of clinical features was performed using Fisher’s exact test. All statistical analyses were conducted using EZR (ver. 1.61, Saitama Medical Center, Jichi Medical University), a graphical user interface for R [22].

## 3. Results

### 3.1. Patient Characteristics

**Figure 1:**
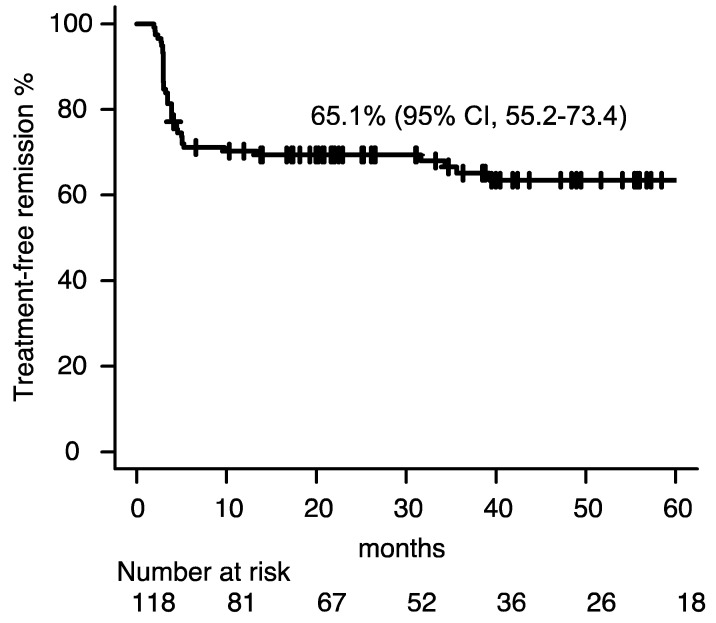
Treatment free remission. Abbreviation; CI, confidence interval.

In total, 118 CML-CP patients discontinued TKIs. The median age was 60 years [interquartile range (IQR): 47–68 years)]; 71 patients were male and 47 were female; 44, 42, and 15 patients had low, intermediate, and high Sokal risk scores, respectively (17 patients had missing data). The frontline TKI was imatinib in 66 cases, dasatinib in 21, nilotinib in 21, and bosutinib in six. The TKIs at discontinuation was imatinib in 43 cases, dasatinib in 37, nilotinib in 37, and bosutinib in five; 83 patients were on a frontline TKI at TKI discontinuation (imatinib, 42; dasatinib, 17; nilotinib, 20 and bosutinib, 4). The duration of the DMR time and median TKI treatment duration was 49.0 months (IQR: 33.2–59.2 months), and 98.8 months (IQR: 62.3–136.8 months), respectively. An estimated 36-month TFR rate was 65.1% [95% confidence interval (CI), 55.2–73.4, Figure 1)] and the median follow-up time for TFR was 39.3 months (IQR, 20.5–62.2). Median white blood cell (WBC) count, neutrophil count, and lymphocyte count at TKI discontinuation were 5800/μL (IQR, 4708–7178), 3210/μL (IQR, 2656–4303) and 1685/μL (IQR, 1131–2181), respectively. Table 1 summarizes the detailed clinical characteristics.

### 3.2. No Clinical Factors Were Identified as Favorable Prognostic Indicators for TFR in Patients with CML-CP

We earlier reported that higher neutrophil counts were favorable prognostic factors for TFR in patients with CML-CP [20], thus we assessed white cell count, neutrophil count, and lymphocyte count at TKI discontinuation according to the using TKIs. Univariate analysis of the clinical properties for TFR (such as sex, age, Sokal risk score, use of frontline TKIs (imatinib or second-generation TKIs), TKIs at discontinuation (imatinib or second-generation TKIs), DMR duration at TKIs discontinuation, total TKIs treatment duration, depth of molecular remission [undetectable measurable residual disease (UMRD) or not] and white blood cell count, neutrophil count or lymphocyte count did not recognize any factor as a substantial prognostic variable for a lesser likelihood of molecular relapse (Table 2). Recently, the introduction of frontline second-generation TKI treatment enhanced the prognosis of patients with CML-CP compared with imatinib, hence we examined the prognostic variables for TFR in CML-CP split into patients with frontline imatinib and frontline second-generation TKIs.

### 3.3. Higher Neutrophil Count at TKI Discontinuation Is a Favorable Prognostic Indicator for TFR in Patients with CML-CP Who Received Frontline Second-Generation TKIs

Sixty-six patients received imatinib as frontline TKI while 52 patients received second-generation TKIs as frontline. Patients who received second-generation TKIs as frontline exhibited longer DMR time (median, 51.0 vs. 46.8 months, *p* = 0.030) and treatment duration (median, 132.0 vs. 62.2 months, *p* < 0.001) than those who received imatinib, while age, sex, Sokal risk score, molecular remission at TKI discontinuation and TFR rate did not vary among the two groups (Table S1).

**Figure 2:**
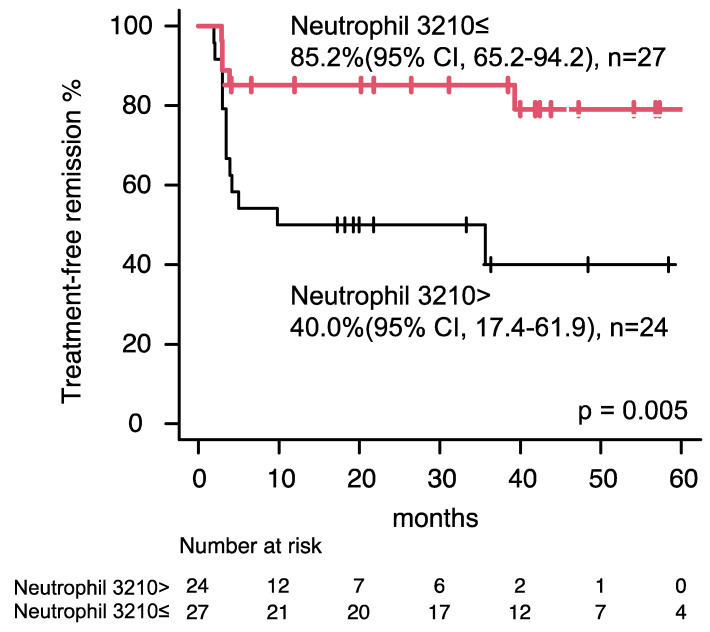
Treatment–free remission in patients who received second-generation tyrosine kinase inhibitors as frontline treatment. Patients with higher neutrophil count (>3210/μL) achieved higher treatment–free remission. Red line indicates Patients with higher neutrophil count (>3210/μL) achieved higher treatment–free remission. Black line indicates patients with lower neutrophil count (≤3210/μL) achieved lower.

Univariate analysis of the clinical properties for TFR displayed extended TKI treatment duration (>98.8 months) was likely to be a favorable prognostic factor for TFR in patients who received imatinib as frontline [HR, 0.444 (95% CI, 0.186–1.060); *p* = 0.067], while did not identify any factor as an important prognostic variable for a lower likelihood of molecular relapse in patients who received imatinib as frontline (Table 3). Whereas, univariate and multivariate evaluation of the clinical characteristics for TFR portrayed higher neutrophil count (>3210/μL) was determined an independent prognostic factor for TFR in a patient who received second-generation TKIs as frontline [HR, 0.235 (95% CI, 0.078–0.711); *p* = 0.010, Table 4 and Figure 2]. We re-evaluated the earlier published data (other cohort) regarding neutrophil count at TKI discontinuation including 53 patients [20]. Patients who received second-generation TKIs as frontline with higher neutrophil count (>2439/μL) favorably acquired TFR [HR, 0.257 (95% CI, 0.080–0.825), *p* = 0.022, Figure S1], while the clinical impact of higher neutrophil count in patients who received imatinib as frontline was insignificant [HR 0.579 (95% CI, 0.174–1.927), *p* = 0.373], in line with the previous study. A higher neutrophil count can be a significant favorable prognostic factor for TFR in patients who received frontline second-generation TKIs supported evidence of the two independent cohorts.

## 4. Discussion

Herein, we have demonstrated a multicenter retrospective study of patients with CML-CP who discontinued TKIs to recognize clinical features linked to sustained TFR. Our outcomes of a TFR rate of 65.1% were comparable to those of previous TKI discontinuation analysis, implying that our patients were a representative cohort [8].

Earlier, we noted a higher neutrophil count at TKIs discontinuation was correlated with the achievement of TFR in patients with CML-CP, whereas a higher neutrophil count at TKI discontinuation can be linked to the achievement of TFR in only patients with CML-CP who received second-generation TKI as the frontline in this research [20]. Remarkably, the magnitude of neutrophil counts did not influence TFR in patients who received imatinib as the frontline in both the previous and the present research, suggesting that higher neutrophil counts may be contributed to only patients with second-generation TKI treatment. Most patients received second-generation TKIs as frontline (31/53 cases, 58.5%) in prior studies, meanwhile, the current study encompassed only 51/118 patients (43.2%, *p* = 0.071). The discrepancy in the clinical impact of neutrophil count for CML-CP between the earlier and the current research may be influenced by the different proportion of patients who received second-generation TKI as frontline.

Neutrophils generally act as anti-bacterial via activation of the innate and adaptive immune system. Recently, tumor-associated neutrophils, particularly antitumor neutrophils have been recognized as a significant component of the cancer microenvironment [23]. The activation of neutrophils triggered *reactive oxygen species* production, causing potent tumor eradication independent from T-cell mediated immune response [24]. T-cell and/or NK cell immune responses partake in the treatment responses or achieving TFR in CML patient, the neutrophil-mediated immune response can also play an important role in CML [25].

Inhibition of the TGF-β pathway causes an accumulation of the antitumor neutrophil, and second-generation TKIs strongly suppress c-Kit and ABL1, a downstream mediator of TGF-β compared with imatinib [26]. The TKIs-activated off-target immunomodulation effects have demonstrated favorable clinical responses [27] and may control residual leukemic cells to attain successful TFR in CML, supported by our retrospective observation. Thus, higher neutrophil counts can be contributed to the patients only by using second-generation TKIs which have strong TGF-β pathway inhibition potential. Whereas, the positive impact of nilotinib which might influence the highest neutrophil counts for TFR was uncertain. Further research is required to clarify the biological mechanism to influence the achievement of TFR by anti-tumor neutrophils.

Our research had several limitations. First, the study was a retrospective observational investigation. Second, there was no assessment of other immune cell fractions (T-cells and NK cells). Third, the study comprised a relatively small cohort (118 patients) compared with other studies (e.g., EUROski) [8]. Fourth, the enrolled patients were all Japanese patients, thus the effects of the neutrophil did not evaluate according to race. 

We conclude higher neutrophil count at TKI discontinuation was related to the achievement of TFR in patients with CML-CP who received second-generation TKI as frontline treatment.

## Figures and Tables

**Table 1:** Patient characteristics.

*n* = 118		No/Median	IQR/%
Age		60	47–68
Sex			
	male	71	60.2%
	female	47	39.8%
Sokal risk score			
	high	15	12.7%
	intermediate	42	35.6%
	low	44	37.3%
	missing	17	14.4%
MR at TKI discontinuation			
	MR4.0	7	5.9%
	MR4.5	53	44.9%
	UMRD	58	49.2%
Frontline TKIs			
	imatinib	66	55.9%
	dasatinib	21	17.8%
	nilotinib	25	21.2%
	bosutinib	6	5.1%
TKI at discontinuation			
	imatinib	43	36.4%
	dasatinib	37	31.4%
	nilotinib	33	28.0%
	bosutinib	5	4.2%
DMR time (months)		49.0	IQR, 33.2–59.2
TKI duration (months)		98.8	IQR, 62.3–136.8
White blood cell (/μL)		5800	IQR, 4708–7178
Neutrophil (/μL)		3210	IQR, 2656–4303
Lymphocyte (/μL)		1685	IQR, 1131–2181

Abbreviations: No, number; IQR, interquartile range; MR, molecular remission; TKI, tyrosine kinase inhibitor, DMR, deep molecular remission.

**Table 2:** Univariate analysis responsible for molecular relapse.

Variables		*n*	HR	95% CI	*p* Value
Age	>60	60	0.652	0.348–1.222	0.182
Sex	male	71	1.309	0.683–2.506	0.417
Sokal risk score	high	15	Ref		
	Int	42	0.576	0.229–1.446	0.240
	Low	44	0.675	0.275–1.657	0.391
	missing	17	0.586	0.186–1.848	0.361
DMR time	>49.0 months	60	1.088	0.583–2.031	0.791
TKI duration	>98.8 months	59	0.663	0.352–1.248	0.202
Frontline TKI	imatinib	66	0.842	0.453–1.567	0.587
TKI at stop	imatinib	42	0.927	0.484–1.776	0.820
MR at stop	UMRD	58	0.667	0.356–1.253	0.208
WBC	>5800	61	1.082	0.581–2.012	0.804
Neutrophil	>3210	58	0.820	0.440–1.529	0.532
Lymphocyte	>1685	58	1.165	0.626–2.167	0.630

Abbreviations: HR, hazard ratio; CI, confidence interval; DMR, deep molecular response, TKI, tyrosine kinase inhibitor; MR, molecular remission; UMRD, undetectable measurable residual disease; WBC, white blood cell.

**Table 3:** Univariate analysis responsible for molecular relapse in patients who received imatinib as frontline treatment (*n* = 67).

Variables		*n*	HR	95% CI	*p* Value
Age	>60	36	0.549	0.234–1.285	0.167
Sex	male	38	1.451	0.608–3.459	0.401
Sokal risk score	high	8	Ref		
	Int	18	0.486	0.130–1.810	0.282
	Low	30	0.682	0.217–2.149	0.514
	missing	11	0.316	0.058–1.728	0.184
DMR time	>49.0 months	39	0.876	0.376–2.037	0.758
TKI duration	>98.8 months	53	0.444	0.186–1.060	0.067
MR at stop	UMRD	33	0.620	0.264–1.456	0.272
WBC	>5800	30	2.127	0.904–5.004	0.084
Neutrophil	>3210	31	1.903	0.812–4.459	0.139
Lymphocyte	>1685	28	1.894	0.817–4.392	0.137

Abbreviations: HR, hazard ratio; CI, confidence interval; DMR, deep molecular response, TKI, tyrosine kinase inhibitor; MR, molecular remission; UMRD, undetectable measurable residual disease; WBC, white blood cell.

**Table 4:** Univariate and multivariate analysis responsible for molecular relapse in patients who received second generation tyrosine kinase inhibitors as frontline treatment (*n* = 51).

			Univariate Analysis			Multivariate Analysis		
Variables		*n*	HR	95% CI	*p* Value	HR	95% CI	*p* Value
Age	>60	24	0.820	0.323–2.080	0.676	0.577	0.201–1.659	0.307
Sex	male	33	1.109	0.416–2.955	0.836			
Sokal risk score	high	7	Ref					
	Int	24	0.672	0.177–2.554	0.560	0.851	0.207–3.493	0.823
	Low	14	0.603	0.135–2.698	0.508	0.704	0.148–3.349	0.659
	missing	6	1.226	0.246–6.108	0.804	2.505	0.390–16.110	0.334
DMR time	>49.0 months	21	1.433	0.567–3.622	0.447			
TKI duration	>98.8 months	6	1.146	0.260–5.049	0.857			
MR at stop	UMRD	25	0.724	0.284–1.846	0.499	0.715	0.233–2.194	0.557
WBC	>5800	31	0.423	0.163–1.097	0.077			
Neutrophil	>3210	27	0.251	0.087–0.724	0.010	0.235	0.078–0.711	0.010
Lymphocyte	>1685	30	0.601	0.237–1.525	0.284			

Abbreviations: HR, hazard ratio; CI, confidence interval; DMR, deep molecular response, TKI, tyrosine kinase inhibitor; MR, molecular remission; UMRD, undetectable measurable residual disease; WBC, white blood cell.

## Data Availability

All data can be accessed by contacting the corresponding author (H.U.).

## Supplementary Materials

The following supporting information can be downloaded at: https://www.mdpi.com/article/10.3390/clinpract14040097/s1, Figure S1: Treatment–free remission according to frontline tyrosine kinase inhibitor (TKI) and neutrophil count at TKI discontinuation in the previous study; Table S1: Characteristics according to the frontline tyrosine kinase inhibitors.
