# Supplementary material for: A Higher Neutrophil Count Is Associated with Favorable Achievement of Treatment-Free Remission in Patients with Chronic Myeloid Leukemia Who Received Second Generation Tyrosine Kinase Inhibitor as Frontline Treatment
Source: Clin Pract. 2024 Jun 21;14(4):1216–24. doi: 10.3390/clinpract14040097 (PMC11270167; doi:10.3390/clinpract14040097)
Supplement: Supplementary file 1 [file clinpract-14-00097-s001.zip › clinpract-2973580-supplementary.pdf]

Table S1. Characteristics according to the frontline tyrosine kinase inhibitors

No/median	Imatinib (n=66)	Second generation (n=52)	P value
Age (median, IQR)	61(49-69)	57(42-67)	0.352
Sex (Male/Female)	38/28	33/19	0.573
Sokal risk (High/Int/Low/Missing)	8/18/29/11	7/24/15/6	0.153
MR ($MR^{4.0}$/$MR^{4.5}$/UMRD)	5/29/32	2/24/26	0.797
DMR time (median, IQR, months)	51.0 (34.1 - 71.8)	46.8 (32.8 - 53.0)	0.030
Treatment duration (median, IQR, months)	132.0 (107.3 - 160.2)	62.2 (52.5 - 72.1)	< 0.001
WBC (median, IQR, /µL)	5400 (4095 - 6585)	6460 (5268 - 7273)	0.012
Neutrophil (median, IQR, /µL)	3156 (2589 - 4160)	3524 (2715 - 4609)	0.245
Lymphocyte (median, IQR, /µL)	1498 (1088 - 1988)	2002 (1318 - 2518)	0.026
TFR rate at 36 months (%, 95% CI)	66.3 (52.6 - 76.8)	63.7 (48.3 - 75.6)	0.583

No, number; IQR, Interquartile range; MR, molecular remission; UMRD, undetectable measurable residual disease; WBC, white blood cell; TFR, treatment-free remission; CI, confidential interval

Figure S1

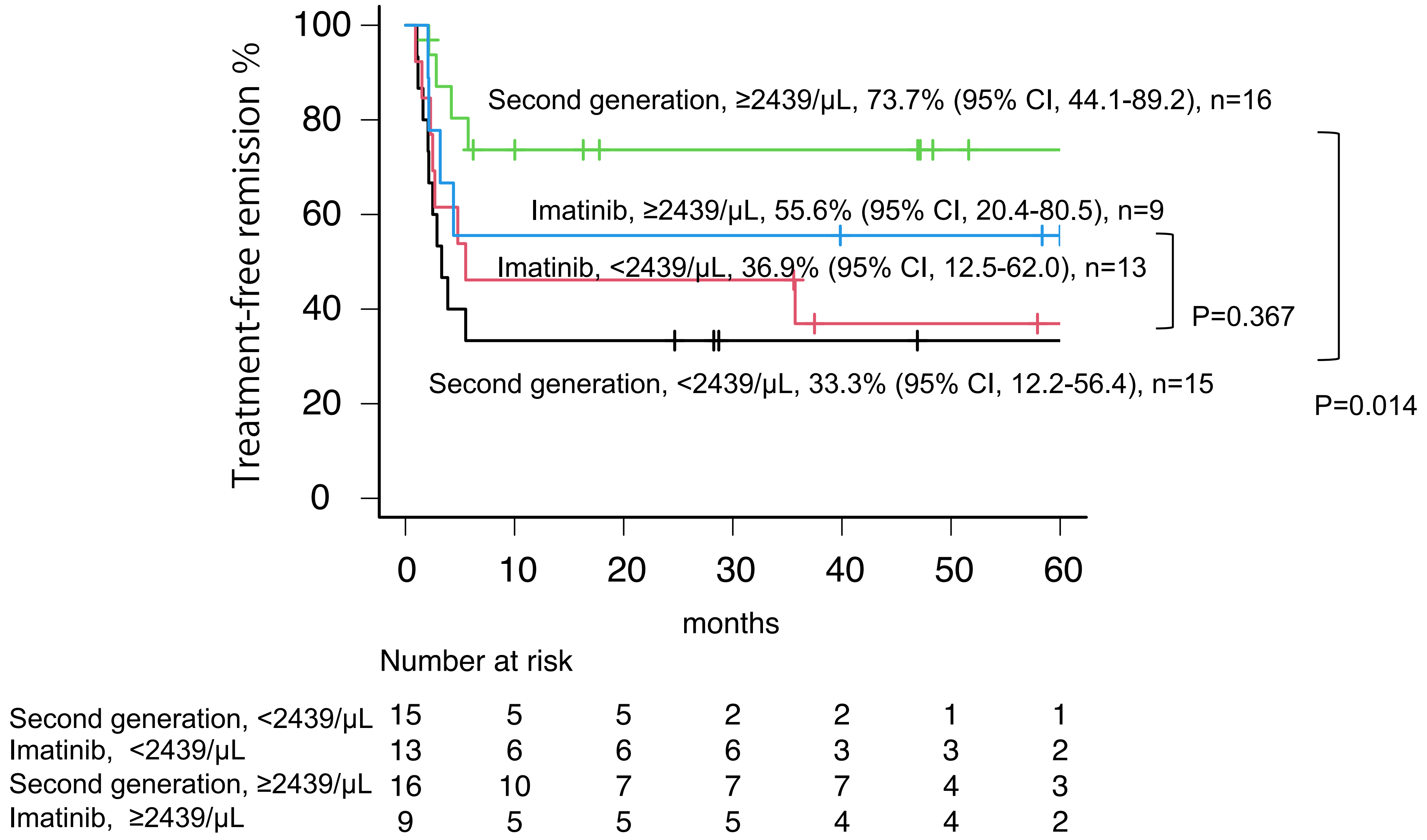

Figure S1. Treatment–free remission according to frontline tyrosine kinase inhibitor (TKI) and neutrophil count at TKI discontinuation in the previous study (Ref. 20).
